# An In-Silico Identification of Potential Flavonoids against Kidney Fibrosis Targeting TGFβR-1

**DOI:** 10.3390/life12111764

**Published:** 2022-11-02

**Authors:** MD. Hasanur Rahman, Partha Biswas, Dipta Dey, Md. Abdul Hannan, Md. Sahabuddin, Yusha Araf, Youngjoo Kwon, Talha Bin Emran, Md. Sarafat Ali, Md Jamal Uddin

**Affiliations:** 1 ABEx Bio-Research Center, East Azampur, Dhaka 1230, Bangladesh; 2 Department of Biotechnology and Genetic Engineering, Faculty of Life Sciences, Bangabandhu Sheikh Mujibur Rahman Science and Technology University, Gopalganj 8100, Bangladesh; 3 Laboratory of Pharmaceutical Biotechnology and Bioinformatics, Department of Genetic Engineering and Biotechnology, Jashore University of Science and Technology, Jashore 7408, Bangladesh; 4 Department of Biochemistry and Molecular Biology, Faculty of Life Sciences, Bangabandhu Sheikh Mujibur Rahman Science and Technology University, Gopalganj 8100, Bangladesh; 5 Department of Biochemistry and Molecular Biology, Bangladesh Agricultural University, Mymensingh 2202, Bangladesh; 6 Department of Genetic Engineering and Biotechnology, School of Life Sciences, Shahjalal University of Science and Technology, Sylhet 3114, Bangladesh; 7 Graduate School of Pharmaceutical Sciences, College of Pharmacy, Ewha Womans University, Seoul 03760, Korea; 8 Department of Pharmacy, BGC Trust University Bangladesh, Chittagong 4381, Bangladesh; 9 Department of Pharmacy, Faculty of Allied Health Sciences, Daffodil International University, Dhaka 1207, Bangladesh

**Keywords:** kidney fibrosis, chronic kidney disease, TGFβR-1, flavonoids, pharmacokinetics, molecular docking, molecular dynamics simulations

## Abstract

Fibrosis is a hallmark of progressive kidney diseases. The overexpression of profibrotic cytokine, namely transforming growth factor β (TGF-β) due to excessive inflammation and tissue damage, induces kidney fibrosis. The inhibition of TGF-β signaling is markedly limited in experimental disease models. Targeting TGF-β signaling, therefore, offers a prospective strategy for the management of kidney fibrosis. Presently, the marketed drugs have numerous side effects, but plant-derived compounds are relatively safer and more cost-effective. In this study, TGFβR-1 was targeted to identify the lead compounds among flavonoids using various computational approaches, such as ADME/T (absorption, distribution, metabolism, and excretion/toxicity) analysis, molecular docking, and molecular dynamics simulation. ADME/T screening identified a total of 31 flavonoids with drug-like properties of 31 compounds, a total of 5 compounds showed a higher binding affinity to TGFβR-1, with Epicatechin, Fisetin, and Luteolin ranking at the top three (−13.58, −13.17, and −10.50 kcal/mol, respectively), which are comparable to the control drug linagliptin (−9.074 kcal/mol). The compounds also exhibited outstanding protein–ligand interactions. The molecular dynamic simulations revealed a stable interaction of these compounds with the binding site of TGFβR-1. These findings indicate that flavonoids, particularly Epicatechin, Fisetin, and Luteolin, may compete with the ligand-binding site of TGFβR-1, suggesting that these compounds can be further evaluated for the development of potential therapeutics against kidney fibrosis. Further, *in-vitro* and *in-vivo* studies are recommended to support the current findings.

## 1. Introduction

Chronic kidney disease (CKD) is a worldwide public health concern, with a growing incidence and frequency of patients requiring replacement therapy [1]. Fibrosis is a major factor in the advancement of practically all types of CKD [2]. Additionally, various other factors, including diabetes, hypertension, infection, ureter obstruction, and genetic alterations, are associated with CKD [3]. In kidney fibrosis, an abnormally large amount of extracellular matrix (ECM) proteins is deposited within the kidney interstitium, glomerular capillaries, and around arterioles in association with inflammatory cell infiltration, tubular epithelial cell loss, and fibroblast accumulation, which can impair the normal physiological functions of the kidney and lead to CKD [2]. Current pharmaceutical interventions for diabetic and non-diabetic CKD patients include angiotensin-converting enzyme inhibitors and angiotensin II receptor antagonists, which offer only modest renoprotection [4]. Thus, there is an unmet need of exploring other potential pharmacological options to treat CKD patients targeting kidney fibrosis.

Transforming growth factor (TGF) is a protein that affects several biological processes, including cell proliferation, differentiation, and immune response. The TGF superfamily consists of 33 members and three TGF isoforms (TGF-1, TGF-2, and TGF-3), with the majority of members being dimeric, secreted polypeptides. TGF, among other things, plays a significant role in renal fibrosis [5,6]. TGF-1 promotes epithelial-to-mesenchymal transition (EMT), which is being more recognized as an important component of renal tissue fibrogenesis [7]. TGFR-1 is also one of the most important receptors in the serine/threonine protein kinase family [8]. TGFR-1 has been shown in studies to contribute to EMT by activating the TGFR-1/Smad signaling pathway. TGFR-1 and its downstream signaling components, such as smad2, smad3, collagen I, collagen IV, and α-SMA, are all increased during EMT [9]. TGFR-1 activation increases profibrotic genes and mediates renal fibrosis, whereas TGF-1 inhibition may minimize kidney injury and fibrosis [10]. As a result, inhibiting the TGF-1/Smad pathway is thought to be a therapeutic strategy for treating renal fibrosis [11]. Diverse therapeutic compounds have been used in kidney fibrosis, but most of the marketed drugs have different side effects; however, plant-based therapeutics, especially flavonoids, have a more reliable history of safer treatment.

From ancient times, plant-derived bioactive phytochemicals have been used as therapeutic agents to treat numerous pathological conditions, including inflammation, oxidative stress, fungal contamination, viral disease, cancer, mutagenic toxicity, neurodegenerative disorders, cardiovascular diseases, and kidney diseases [12,13,14,15,16,17,18,19]. Although alkaloids, flavonoids, terpenoids, and lignans, the major classes of bioactive phytochemicals, are one of the most promising and alternative options in new drug discovery as well as development, flavonoid-based phytochemicals have shown different biological activity towards disease conditions, including kidney disease [20,21,22] and liver disease [23,24]. Epigallocatechin-3-gallate is one of the major flavonoid phytochemicals that is closely associated with the improvement of kidney fibrosis [20]. Additionally, Luteolin was shown to be effective against nephropathy in streptozotocin (STZ)-induced diabetic rats [25]. Recently, resveratrol showed the most significant effects against various kidney diseases along with minimizing mortality risk [26,27]. In addition, (-)-Epicatechin possesses beneficial properties in the rat kidney [28]. Ethyl acetate extract of *Coreopsis tinctoria* Nutt has enriched potential flavonoids and possesses anti-fibrotic activity in STZ-induced diabetic rats via targeting the TGFβ-1 [29,30]. Consequently, quercetin is a major flavonoid compound, which directly suppresses the overexpression of TGFβ-1 and ameliorates the severity of diabetic nephropathy in STZ-induced diabetic rats [31]. However, the functional role of flavonoids targeting TGFβR-1 in kidney fibrosis has not yet been explored.

**Figure 1:**
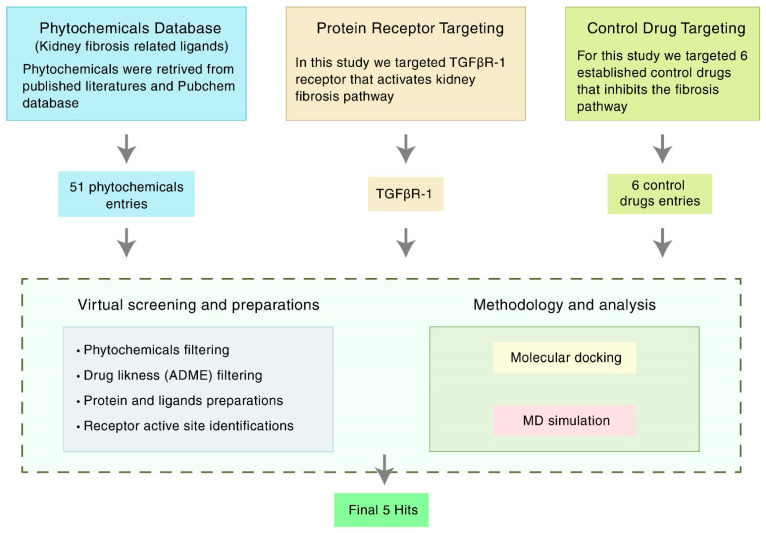
Schematic overview of the strategies targeting TGFβR-1 (PDB ID: 6B8Y) signaling. TGFβR-1 = Transforming growth factor beta receptor 1; MD simulation = Molecular dynamics simulation; ADME = Absorption, distribution, metabolism, and excretion.

Considering the emerging need for a therapeutic option to treat kidney fibrosis, we performed an In-Silico identification of potential natural flavonoids targeting TGFβR-1 (Figure 1). In this present In-Silico workflow, we selected 51 natural flavonoids from the published literature, where phytochemicals have a potential role against kidney disease, and subsequently, we chose the best 31 compounds based on their pharmacokinetic properties, including ADMET (absorption, distribution, metabolism, elimination, and toxicity). Additionally, to find out the best ligands (i.e., phytochemicals), we conducted a molecular docking study against the targeted protein TGFβR-1 (PDB ID: 6B8Y) and found the best five compounds based on their docking score. The top five compounds with protein receptor interactions were also analyzed via the BIOVIA Discovery Studio Visualizer, which produced more positive results than the control. Finally, the Desmond (Schrödinger Release 2020-3) paid simulator package was applied to validate the “protein–ligand complex” files and produced more acceptable results.

## 2. Materials and Methods

### 2.1. Receptor and Ligand Selection

For the docking study, phytochemicals with potential anti-kidney fibrosis effects were considered. Heterobicyclic inhibitors of TGFβR-1 [32] and drugs, such as Losartan, Sitagliptin, Vildagliptin, Saxagliptin, Linagliptin, and Alogliptin, were considered as the standard references for the study.

### 2.2. Compound Screening and ADMET Prediction

In clinical trials, QikProp (Schrödinger Release 2020-3, Schrödinger, LLC, New York, NY, USA, 2020) finds lead compounds that perform better in terms of ADMET. QikProp is a powerful ADMET prediction tool for pharmaceutical companies. The number of attributes that extend outside the 95 percent range of similar values for recognized medications is indicated by the #stars (Supplementary Table S1). The researchers looked at 51 bioactive ligand molecules linked to kidney fibrosis. The chemicals were all derived from a variety of published sources.

### 2.3. Protein Preparation

According to the RCSB Protein Data Bank (PDB) (https://www.rcsb.org/, accessed on 24 July 2021) database information, TGFβR-1 has five existing inhibitors and the PDB ID 6B8Y shows the highest resolution and highest reliability [33]. The crystal structure of 6B8Y was downloaded using the RCSB PDB and preprocessed using Protein Preparation Wizard (Maestro Desmond version 12.5). The default parameters of assigned bond orders, CCD database, addition of hydrogens, creation of zero-order bonds to metals, creation of disulfide bonds, and filled missing side chains and loops were used by Prime (Maestro Desmond version 12.5). Additionally, the missing cap termini was fixed, and waters beyond 5 was deleted, as well as generating heat states of pH 7.0 +/− 2.0 were used by Epik (Maestro Desmond version 12.5). The H-bond was assigned in PROPKA with pH level 7.0 and the degradation was limited with the coverage heavy atom to RMSD 0.30 Å by using the refine tab with force field of OPLS3e.

### 2.4. Ligand Preparation

The three-dimensional structure SDF file format of the phytochemicals and the standard drugs were downloaded from the open-source PubChem database (https://pubchem.ncbi.nlm.nih.gov/, accessed on 24 July 2021). LigPrep (Schrödinger Release ver. 2020-3) was used to prepare the ligand structures for this study. At the time of preparing the ligands, the default ligprep parameters were used with a standard pH of 7.0 to (+/−) 2.0, a maximum number of conformers per structure was 32, and RMSD value was 1.0 Å. Additionally, a minimization was performed using the OPLS3e force field and the Epik ionizer.

### 2.5. Active Site (ASs) Identification and Receptor Grid Generation

After the protein preparation of 6B8Y was performed, the possible binding site of the TGFβR-1 domain was developed using the Schrödinger Sitemap (Maestro version 12.5). The SiteMap in Maestro is a method for locating putative binding sites on a protein for small-molecule ligands. It plots and scores regions on the protein surface that are likely to contain a ligand. The new SiteMap is a development of Maestro’s initial SiteMap facility (formerly known as hppmap) [34,35]. Site mapping works similarly to Goodford’s GRID algorithm [36], as it did in the original approach [37]. There are three processes to calculating a SiteMap. However, to establish the active sites of 6B8Y protein, a grid is first created by the Receptor Grid Generation tool with Van der Waals residue (scaling factor of 1.0 Å and partial charge cutoff 0.25 Å), and the points are then organized into groups based on several criteria. Second, the sites are mapped on a different grid to provide files for map visualization. Finally, properties are accessed, and sites are documented in a Maestro-friendly format. An impact job is used to complete each level. The binding pocket with the highest SiteScore and druggability score (DScore) was chosen for molecular docking investigations.

SiteScore = 0.0733 *n*^1/2^ + 0.6688 *e* − 0.20 *p*, where n is the number of site points (limited at /100), e is the enclosure score, and *p* is the hydrophilic score, which is set at 1.0 to restrict the impact of hydrophilicity in charged and highly polar sites [38].

DScore uses the same properties as SiteScore but different coefficients:

DScore = 0.094 sqrt *n*^1/2^ + 0.60 *e* − 0.324 *p*

The hydrophilic score is not capped in DScore. This is one of the fundamental differences between “difficult” and “undruggable” targets and “druggable” targets [34]. Because these are independent and occasionally competing purposes, the use of different functions for binding-site identification and druggability classification is appropriate. For example, ligands that have nanomolar, and even subnanomolar, affinity for the PTP1B phosphate pocket [39]. However, these extremely active ligands are not drug-like and contain charge configurations similar to those of the natural phosphate substrate. Although such a region can bind ligands tightly, it should not be classified as druggable by the SiteMap [34].

### 2.6. Molecular Docking and Visualization

After the completion of extra precision (XP) molecular docking by Maestro (v12.5), every protein–ligand complex PDB structure was extracted from the docked post-viewing file for their post-docking visualization analysis of the non-bond interaction and the hydrophobicity. The Discovery Studio Visualizer (v.21) was used to perform post-docking visualization on the protein–ligand complex structures. Furthermore, the non-bonded and non-covalent bonded interactions were also carried out by the Discovery Studio Visualizer [40].

### 2.7. Binding Free Energy Calculation by Using Prime/MM-GBSA Approach

MMGBSA is known as the molecular mechanics-generalized Born surface area, which can be performed to calculate ligand binding free energies and ligand strain energies for a set of ligands and a single receptor. After completing the site-specific molecular docking, the MMGBSA was conducted by utilizing the Prime model of Schrödinger suite 2020-3, which analyzes the relative binding free affinity of the control and candidate ligands against the selected 6B8Y protein receptor.

### 2.8. Molecular Dynamics (MD) Simulations

The possible ligand compounds’ binding consistency to the targeted protein AS was determined using 200 ns MD simulations [41]. The Desmond (v12.5) molecular dynamic simulation of protein–ligand complex structures was used to investigate the thermodynamic stability of receptor–ligand complexes [42]. For this framework, a pre-determined TIP3P water approach was formulated to maintain a particular volume with the orthorhombic periodic bounding box shape with a distance of 10 Å. Appropriate ions, such as O+ and 0.15 M salt (Na^+^ and Cl^−^), were chosen for electrically neutralizing the framework and randomly placed inside the solvent system. The system framework was reduced and relaxed by using the default protocol implemented by using force field OPLS3e within the Desmond module after the construction of the solvency protein system with a ligand complex [43]. The temperature of NPT assemblies that used the Nose–Hoover temperature combining technique and the isotropic technique was kept at 300 K and one atmospheric pressure (1.01325 bar), with 50 ps capture periods with an energy of 1.2 ps.

#### 2.8.1. Simulation Trajectory Analysis

Schrödinger’s maestro v12.5 was used to create the molecular dynamic simulation visualizations. The Simulation Interaction Diagram (SID) from Desmond modules was used to evaluate the simulation event and verify the MD simulation’s quality. The root-mean-square deviation (RMSD), protein–ligand contact (P-L), and the hydrogen bond interaction were used to determine the stability of the protein–ligand complex structure.

#### 2.8.2. RMSD Analysis

RMSD is the average distance caused by the dispossession of a single atom over time [42]. RMSD of protein structural atoms, such as Cα, backbone, sidechain, and heavier atoms, is tallied first, followed by the RMSD of protein fit ligand atoms from all time frames, which is aligned and measured against the reference time (200 ns). The *RMSD* of an MD simulation with a period of *x* can be determined by using the equation below (Equation (1)).

(1) $${\mathrm{RMSD}}_{x}=\sqrt{\frac{1}{N}\;\sum_{i=1}^{n}\left(r{’}_{i}\left(t_{x}\right)\right)-r_{i}{\left(t_{ref}\right)}^{2}}$$

*N* is the number of atoms chosen, *t_ref_* denotes the reference time, and *r*′ denotes the position of the chosen atom in the system *x* after superimposing the reference system’s point.

## 3. Results

### 3.1. Compound Screening and ADME/T

The phytochemical properties of a molecule should fall within the following range, according to “Lipinski’s rule of five”, H-bond donors 5, H-bond acceptors 10, log P 5, and molecular weight 500 kDa. Furthermore, a substance is more likely to have poor absorption if two or more of the characteristics are not fulfilled [44]. Only 30 of the 51 phytochemicals analyzed met the criteria for further analysis (Supplementary Table S1).

TGFβR-1 structure was optimized for further investigation, and ligand molecules were eliminated based on ADME/T findings. However, 8 bioactive compounds did not display ADME/T properties, and 13 compounds did not achieve their minimum ADME/T recommended values (Supplementary Table S1), which is why we conducted our further study with the remaining 30 of 51 compounds (Supplementary Table S2).

### 3.2. AS Identification and Receptor Grid Generation

**Figure 2:**
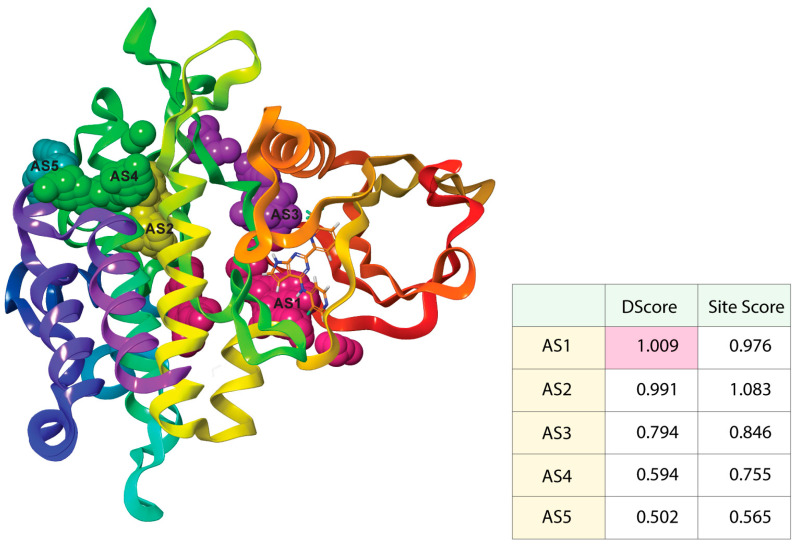
Active sites with the corresponding binding position of TGFβR-1. Ball shape with pink, yellow, purple, green, and blush color represent AS1, AS2, AS3, AS4, and AS5, respectively, with respect to their binding site position of TGFβR-1.

In an enzyme, the binding site is a specific area of the protein where amino acid residues precisely form a temporary bond with the substrate [45]. The predicted receptor domain binding sites are needed for molecular docking studies. As the 6B8Y crystal structure with ligands is unavailable in the database, these active site residues need to be predicted. After the analysis of the 6B8Y protein, five AS (AS1, AS2, AS3, AS4, and AS5) were generated (Figure 2). All the AS displayed hydrogen-bond acceptor map, hydrogen-bond donor and hydrophobic and hydrophilic map, metal-binding map, and surface map were good. The druggability of a binding site was measured by its DScore, and the higher the DScore, the better the site [35,46]. As the AS1 showed the highest DScore, this binding site was chosen for molecular docking studies and a grid was generated across the site by Glide application (Maestro v12.5). Glide (Grid-based Ligand Docking with Energetics) seeks out advantageous interactions between ligand and receptor molecules. Several different sets of fields depict the structure and features of the receptor on a grid, providing a progressively more accurate scoring of the ligand [47].

### 3.3. Molecular Docking and MMGBSA Analysis

Molecular docking is a computer-aided drug design technique, which uses certain algorithms to allocate affinity scores according to ligand positions in the target pocket. The highest binding affinity is the lowest docking score, which implies that the highest binding compound complex spends more time in contact [48].

5-Hydroxy-3,6,7,8,3′,4′-hexamethoxyflavone (C-01), (-)-Epicatechin (C-06), Resveratrol (C-09), Fisetin (C-18), and Luteolin (C-29) returned the best binding affinity score of all (Table 1). We also considered the six FDA-approved drugs of Losartan, Sitagliptin, Vildagliptin, Saxagliptin, Linagliptin, and Alogliptin as the control drugs, but Linagliptin (C-32) showed the best docking score (Supplementary Table S3) and we selected this drug as our reference drug for this study.

The 5 final selected compounds among the 31 phytochemicals (Table 1) had a docking score range from −13.0 to −7.0 kcal/mol.

Molecular docking was also evaluated using MMGBSA-free restricting vitality, which is identified with the post-scoring strategy for TGFβR-1 (PDB ID: 6B8Y) target. The docking correctness was checked by inspecting the lowest energy poses predicted by the scoring functions (Supplementary Table S4: Part 1 and 2). The greater the negative free energies of the MMGBSA binding value, the stronger the binding (Table 1). In the AS1 binding site of 6B8Y, C-18 had the highest negative binding energy of approximately 51.63 kcal/mol, C-06 had a predictive binding energy of −48.19 kcal/mol, and C-32 had a binding energy of −41.57 kcal/mol.

### 3.4. Analysis of Protein–Ligand Interactions

**Figure 3:**
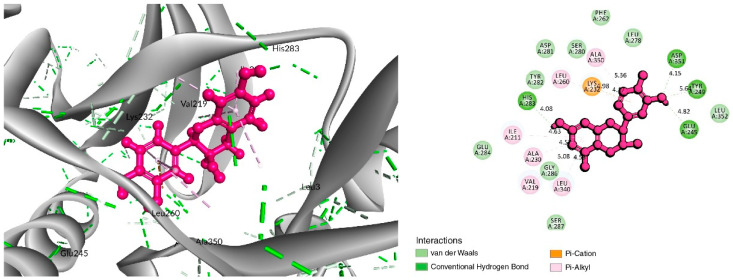
Interaction between Epicatechin (pink color) and 6B8Y. The **left** figure depicts the 3D complex protein–ligand interactions, while the **right** figure depicts the 2D complex protein–ligand interactions.

The BIOVIA Discovery Studio Visualizer tool (v21) was used to observe the interactions between the selected five ligands and the desired protein. With the TGFβR-1 protein, C-06 was found to form several conventional and carbon–hydrogen bonds. Four conventional hydrogen bonds were found to form at the positions of Asp351 (4.15 Å), Tyr249 (5.64 Å), Glu245 (4.82 Å), and His283 (4.08 Å) (Figure 3 and Table 2). Six Pi–Alkyl bonds were also found at the positions of Ala350 (536 Å), Leu260 (4.98 Å), Ile211 (4.63 Å), Ala230 (4.57 Å), Val219 (5.08 Å), and Leu340 (4.58 Å), and a Pi–Cation bond was also observed during the interaction of C-06 in the position of Lys232 (4.17 Å).

**Figure 4:**
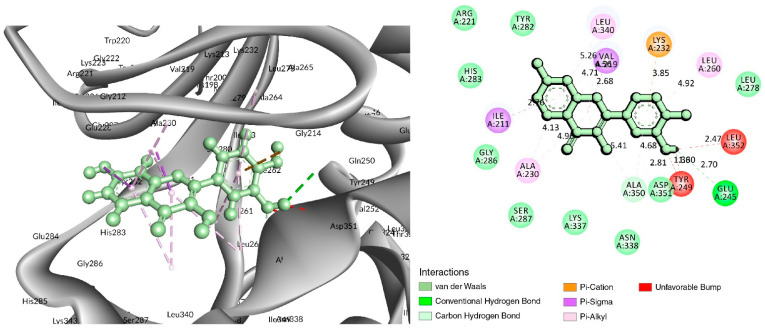
Interaction of 6B8Y with Fisetin (cyan color). The **left** figure depicts a 3D complex protein–ligand interaction, while the **right** figure depicts a 2D complex protein–ligand interaction.

In the case of the C-18 phytochemical, two conventional hydrogen bonds were found at the positions of Glu245 (2.7 Å) and Ala350 (2.81 Å). Three Pi–Alkyl bonds were found at Ala230 (4.13 Å), Leu240 (4.71 Å), and Leu260 (4.92 Å). Two Pi–Sigma and a Pi-Cation bond were also found at the positions Ile211 (2.76 Å), Val219 (2.68 Å), and Lys232 (3.85 Å), respectively (Figure 4 and Table 2).

**Figure 5:**
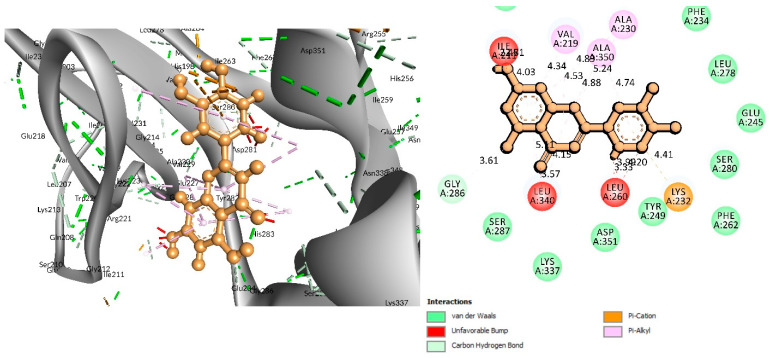
Interaction of 6B8Y and Luteolin (maroon color). The **left** figure depicts a 3D complex protein–ligand interaction, while the **right** figure depicts a 2D complex protein–ligand interaction.

For the C-29 phytochemical, one carbon–hydrogen bond was found at Gly286 (3.61 Å), in addition to three Pi–Alkyl, two Pi–Sigma, and one Pi–Cation bond also being found at, respectively, the Ala230 (4.13), Leu340 (4.71), Leu260 (4.92 Å), Ile211 (2.76 Å), Val219 (2.68 Å), and Lys232 (3.85 Å) positions (Figure 5 and Table 2).

**Figure 6:**
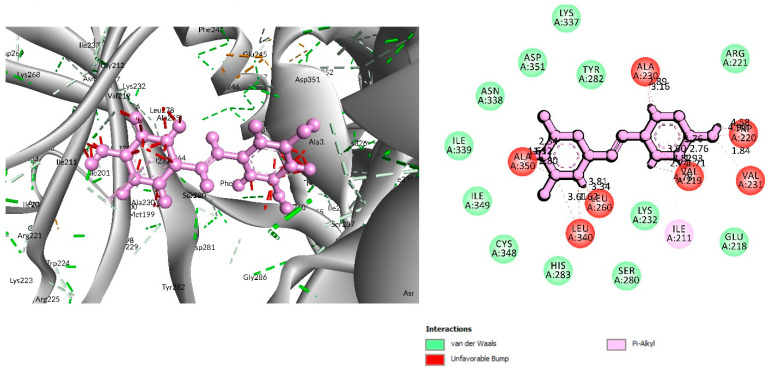
Interaction between 6B8Y and Resveratrol (light purple color). The **left** figure illustrates a 3D complex protein–ligand interaction, while the **right** figure represents a 2D complex protein–ligand interaction.

**Figure 7:**
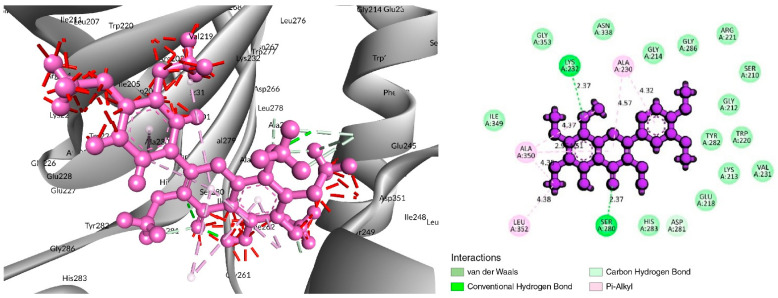
Interaction of 6B8Y and 5-Hydroxy-3,6,7,8,3′,4′-hexamethoxyflavone (navy-blue color). The **left** figure illustrates a 3D complex protein–ligand interaction, while the **right** figure represents a 2D complex protein–ligand interaction.

The interaction study of phytochemical C-09 found one Pi–Alkyl bond at the position of Ile211 (4.78 Å) (Figure 6 and Table 2). In the study of the C-01 phytochemical, there were two conventional hydrogen bonds at the positions of Lys232 (2.37 Å) and Ser280 (2.37 Å). Two carbon–hydrogen bonds were also found at the position of Ala350 (2.95 Å) and also other Pi–Alkyl bonds were found at the Ala230 (4.57 Å) position (Figure 7 and Table 2).

### 3.5. MD Simulations Analysis

Using MD simulations, it is possible to confirm the stability of the protein–ligand complexes in an artificial environment. To establish the binding consistency of the candidate ligand molecules to the target TGFβR-1 protein AS1, 200 ns MD simulations were performed on the protein–ligand complex structures. Remarkably detailed descriptions of these MD simulation results are provided in terms of RMSD, intramolecular hydrogen bonding (Intra HB), and protein–ligand interaction analysis (P–L contact).

#### 3.5.1. RMSD Analysis

**Figure 8:**
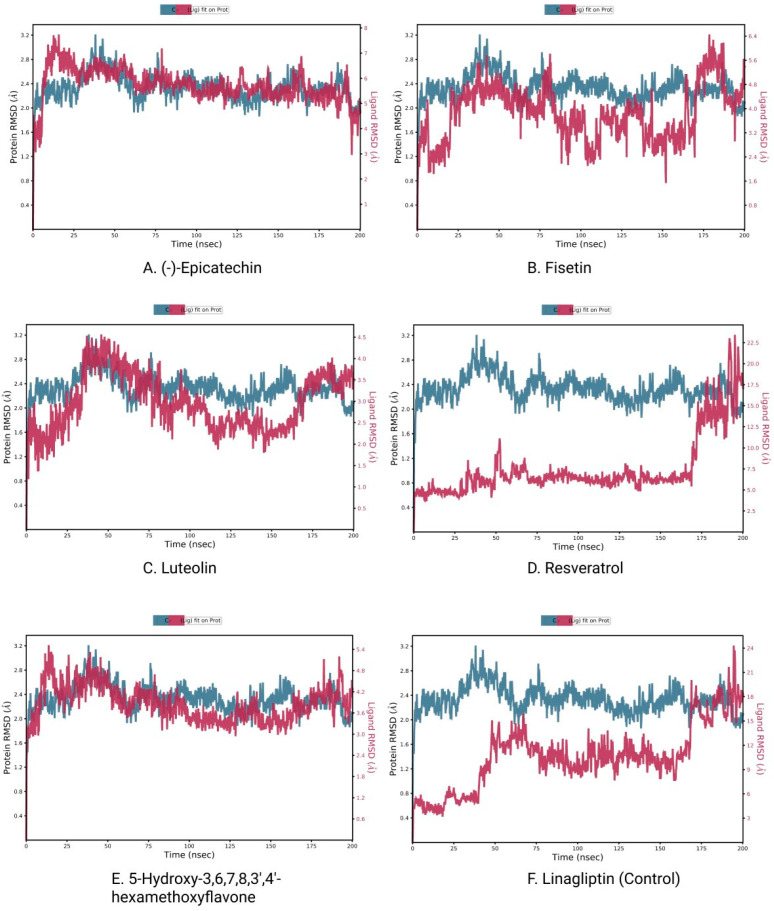
RMSD plots of protein–ligand interactions. (**A**) (-)-Epicatechin, (**B**) Fisetin, (**C**) Luteolin, (**D**) Resveratrol, (**E**) 5-Hydroxy 3,6,7,8,3′,4′-hexamethoxyflavone, and (**F**) Linagliptin.

The measurement of the average displacement of atoms from one reference frame to another was conducted using RMSD. The acceptable RMSD range is from 0.01 to 3.5 Å micrometers. Figure 8 depicts the RMSD progression of a protein (left *Y*-axis) and a ligand (right *Y*-axis). The RMSD for the (-)-Epicatechin, Fisetin, Luteolin, Resveratrol, 5-Hydroxy-3,6,7,8,3′,4′-hexamethoxyflavone, and the control drug Linagliptin was determined after all protein frames were aligned on the reference frame backbone (Figure 8A–F). The RMSD of the protein found the stability of the four compounds except for the compound Resveratrol (the average value changes from the TGFβR-1 backbone to (-)-Epicatechin, Fisetin, Luteolin, and 5-Hydroxy-3,6,7,8,3′,4′-hexamethoxyflavone) within the range of 0.8–3.2 Å, where the value of Resveratrol changed from 0.6 Å, which was lower than the desired range, indicating the large conformational change of the protein. At the beginning of 0–27 ns, it was found that the fluctuation increased for (-)-Epicatechin and 5-Hydroxy-3,6,7,8,3′,4′-hexamethoxyflavone and decreasing for Fisetin and Luteolin. The state of equilibration was achieved after 30 ns for (-)-Epicatechin and 5-Hydroxy-3,6,7,8,3′,4′-hexamethoxyflavone, but the fluctuation decreased again after 90–170 ns for Fisetin and Luteolin, but maintain an average range distance of 0.8–3.2 Å. As a result, we should take into account the fact that the fluctuations will be optimized throughout the course of the long simulation run.

The RMSD of a ligand reflects how stable it is in relation to the protein and its binding pocket. When the protein_-ligand complex is first aligned on the reference protein backbone and then the RMSD of the ligand heavy atoms is measured, the RMSD of the ligand is presented in the plot. If the observed values are significantly greater than the RMSD of the protein, the ligand has very likely diffused away from its initial binding site [49]. In that scenario, we calculated the RMSD of compounds to determine the stability of the chosen compounds in comparison to the control compounds. Here, the observation of the compound found optimal RMSD for Resveratrol, and all other compound distances are significantly larger (>0.8 nm) than the RMSD of the control compound; therefore, the compound can diffuse away from its initial binding site.

#### 3.5.2. Protein–Ligand Contacts

**Figure 9:**
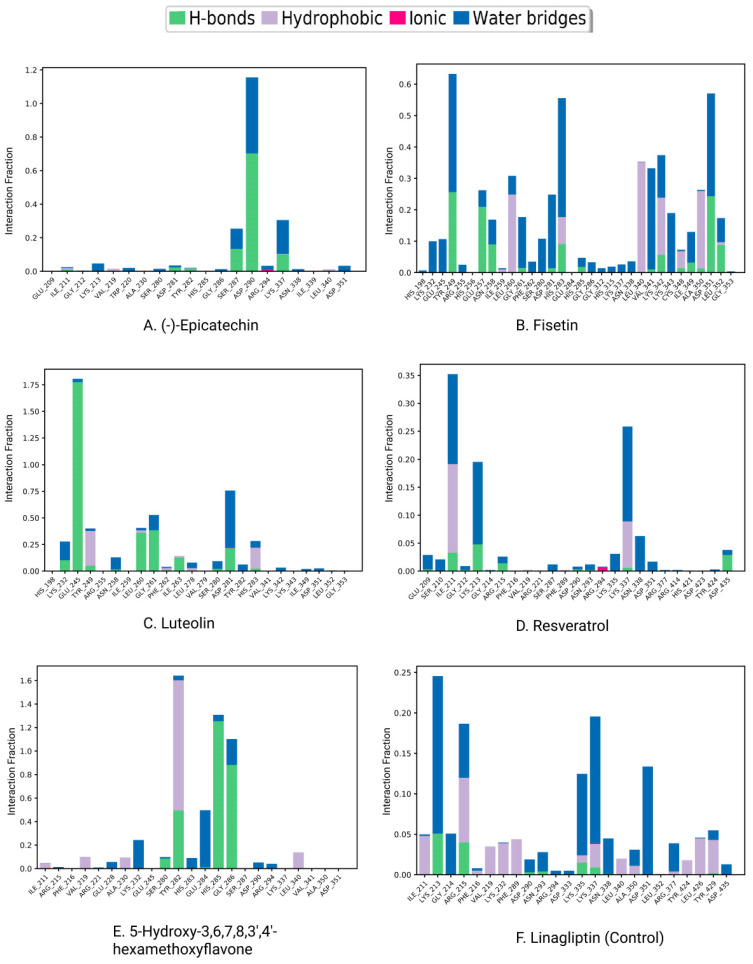
Protein–ligand contacts and ligand–protein contact (**right**) of the (**A**) (-)-Epicatechin, (**B**) Fisetin, (**C**) Luteolin, (**D**) Resveratrol, (**E**) 5-Hydroxy-3,6,7,8,3′,4′-hexamethoxyflavone, and (**F**) Linagliptin (control) at 200 ns of the simulation time.

**Figure 10:**
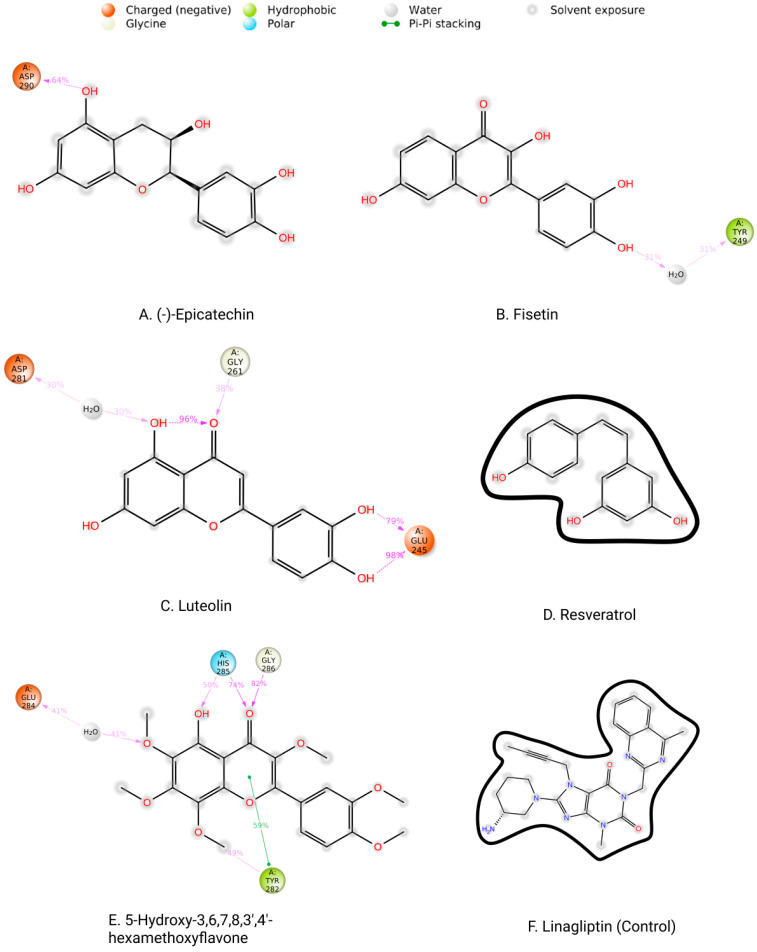
Two-dimensional interactions of the ligand–protein complex. (**A**) (-)-Epicatechin, (**B**) Fisetin, (**C**) Luteolin, (**D**) Resveratrol, (**E**) 5-Hydroxy-3,6,7,8,3′,4′-hexamethoxyflavone, and (**F**) Linagliptin (control).

Protein interactions with the ligand may be seen throughout the 200 ns simulation. These interactions can be categorized and summarized by type, as shown in the graph above. Hydrogen bonds, hydrophobic interactions, ionic interactions, and water bridges are the four forms of protein–ligand interactions. The ‘Simulation Interactions Diagram’ panel can be used to look at the subcategories of each interaction type. The stacked bar charts are normalized throughout the journey. The stacked bar charts for (-)-Epicatechin indicate an interaction fraction value (IFV) of 1.1 Å at residue Asp 290, which makes contact via hydrogen and water bridge bonds, indicating that the specific interaction was maintained for over 100% of the simulated time (Figure 9A). The IFV detected a maximum of 1.75 Å (Glu 245) that was formed by the hydrogen and water bridge bond, as well as 0.8 Å at Asp 281 that was produced by the hydrogen and water bridge bond, indicating being maintained above 100% and 80%, respectively, for the compound Luteolin (Figure 9C). Furthermore, for the compound 5-Hydroxy-3,6,7,8,3′,4′-hexamethoxyflavone, the IFV value is a maximum of 1.6 Å at the position of Tyr, which was formed by a hydrogen, hydrophobic, and water bridge bond and IFV also found 1.2 Å and 1.1 Å at His 285 and Gly 286, respectively, both having been formed by a hydrogen and water bridge bond, indicating a maintenance of over 100% (Figure 9E). Compared with the three compounds and the control, the other two compounds maintained a low IFV and formed multiple hydrogens, hydrophobic, water bridge bonding, and ionic bond interactions. In the case of Fisetin, the IFV is low at the position of Asp 281 until Asp 251 but maintained an optimized IFV value from Tyr 249 to His 283 (Figure 9B). For the compound Resveratrol, the IFV is maintained very low at the position of Lys 213 and Lys 337, and the position of Ile 211 has an optimized IFV value (Figure 9D). The compound formed the minimum hydrogen bond with the desired protein, and therefore the stability of the compound should be hindered. In the case of Linagliptin, it was found to form multiple interactions with the same residue within the same atom of the ligand due to the sidechain having more than one H-bond donor feature (Figure 9F). Figure 10 shows interactions between the ligand and protein that occur for more than 30% of the simulation time for the selected compounds in a 2D format. Because some residues may have several contacts of the same type with the same ligand atom, interactions with >100% are feasible. In the Asp (290) residue, the (-)-Epicatechin displays 64% interactions from 0.00 to 200 ns of the simulation period. Fisetin has a 31% interaction in the Tyr (249) residue, while Luteolin and 5-Hydroxy-3,6,7,8,3′,4′-hexamethoxyflavone have several interactions, with the largest interaction being 98% with Glu (245) and 82% with Gly (286). Resveratrol and Linagliptin did not show any interactions over the simulation time.

## 4. Discussion

Fibrosis is a common feature of all kinds of chronic kidney disease. TGF-β signaling plays a crucial role in cellular physiology. However, its deregulation contributes substantially to the development of renal fibrosis [50]. Pharmacological intervention to the deregulated TGF-β signaling could offer a promising approach in the management of renal fibrosis. Accumulating evidence suggests that phytochemicals, particularly flavonoids, can protect against inflammation or injury in kidney tissues [20]. However, the antifibrotic potential of flavonoids targeting TGFβR-1 in the kidney has yet to be explored. In this paper, a combined virtual screening and molecular simulation approach identified a total of five compounds that exhibited a higher binding affinity for TGFβR-1.

In recent years, In-Silico drug design has attracted attention due to its ability to accelerate the development of new drugs by analyzing the results of bioactivity profiling, molecular modeling, post-docking screening, MDS, and predictive analytics of noble compounds against various diseases [51,52]. Our study has screened 52 flavonoid compounds to evaluate their inhibitory activity against the TGF-β receptor 1 (PDB ID 6B8Y) to search for a potential drug candidate for the treatment of kidney fibrosis. Initially, molecular docking was performed to identify the best five ligand compounds based on their binding affinity score and selected one FDA-approved drug used as the control. At first, in our study, we selected 51 flavonoid compounds; however, after chemical and ADMET screening, 31 flavonoids were approved for the subsequent analysis. ADMET assessment is a cost-effective strategy for reducing drug development costs while providing “fact checks” and secondary complimentary views for high-performance imaging techniques [53,54]. Our study conducted the qikprop application of the Schrödinger package software to complete the ADMET profiling.

Molecular docking is a technique for assessing how two or more molecules will bind with the highest compositional confirmation and the lowest binding affinity. Drug candidates that delivered the most significant and stable score were selected using the Maestro application, which utilizes molecular docking to assign a score. The molecular docking study for the selected five flavonoid compounds and control drug with the TGF-β receptor 1 (PDB ID 6B8Y) reported the docking affinity of the control drug Linagliptin (PubChem CID 10096344) was −9.074 kcal/mol. Among the selected flavonoid compounds, (-)-Epicatechin (PubChem CID 72276) possessed the best binding affinity of −13.585, in which Fisetin (PubChem CID 5281614) had −13.177 and Luteolin (PubChem CID 5280445) had −10.506; all docking affinity results with their chemical structure are depicted in Table 1. Furthermore, the Discovery Studio Visualizer tool (v.21), an effective visualizer tool for drug discovery, was used to represent the post docking receptor–ligands interactions with their animated and 2D structures.

MDS is a versatile technique that analyzes biomolecular interactions and the contact between the arrangement and activity of proteins to aid in modern drug discovery and performance data from dynamic trajectory analysis [55]. Our study conducted the Desmond application of Schrödinger package software (Schrödinger Release 2020-3) to run Molecular Dynamic simulation (MDS) with the selected physiological and physicochemical parameters. This simulation trajectory of the simulation tool has also been used to perfectly analyze the root-mean-square deviation (RMSD), the radius of gyration (Rg), hydrogen bond number, and solvent accessible surface area (SASA) [56]. The root-mean-square deviation (RMSD) of the selected TGF-β receptor 1 protein backbone was used to evaluate the protein structure’s reliability and identify conformational changes; the lower value indicates the most stable compounds [57]. RMSD values of less than 1.5 Å are typically indicative of greater consistency in docking since RMSD values over 1.5 Å typically indicate the average binding positions. In our study, the RMSD values of the protein–ligand interactions were within an appropriate range, namely, the average mean values of 2 (the lowest value for CAP is approximately 0.8, and maximum values of 3), suggesting a better docking position and no disruption of the protein–ligand structure (Figure 8).

On the other hand, the protein–ligand contacts, classified as the hydrogen bonds, hydrophobic interactions, ionic interactions, and water bridges, have a significant role in both protein–ligand complexes and their molecular recognition. All three protein-ligand complexes remained stable throughout the simulation trajectory, indicating that the complexes are rigid (Figure 9). Luteolin and 5-Hydroxy-3,6,7,8,3′,4′-hexamethoxyflavone were found to have strong hydrogen bonds, hydrophobic bonds, and water bridges with protein residues. Fisetin also showed strong hydrogen bonds, hydrophobic bonds, and water bridges with the protein structure. Furthermore, the other drugs had typical molecular interactions with the protein residues.

In the present In-Silico investigation, there was no available clinical research that provided clinical proof of these selected flavonoid compounds for inhibiting TGFβR-1 expression as the target for kidney fibrosis treatment. This research study concentrated on a novel approach to treat kidney fibrosis, indicating that additional wet lab and clinical studies might be necessary to authenticate these drug-like natural aromatic flavonoid compounds targeting TGFβR-1. This means that, after the validation of the activity in in vitro and in vivo modeling, these selected aromatic potential bioactive flavonoid compounds can be used as an alternative treatment option for kidney fibrosis.

## 5. Conclusions

With the increasing prevalence and incidence of kidney fibrosis worldwide, novel and viable therapeutic options are essential to ease the burden of this deadly disease. Notably, TGFβR-1 is one of the most important receptors in the serine/threonine protein kinase family that has been shown to contribute to EMT by activating the TGFβR-1/Smad signaling pathway leading to renal fibrosis. Considering this, in our present study, an In-Silico screening of prospective flavonoids targeting TGFβR-1 as a therapeutic approach to treat kidney fibrosis was conducted. We assembled a library of 51 flavonoids based on their pharmacokinetic properties. The virtual ADME/T analysis screened a total of 31 flavonoids with drug-like properties. In molecular docking analysis, a total of five compounds, notably Epicatechin, Fisetin, and Luteolin, exhibited higher binding affinities with the TGFβR-1 protein, which were compared to the standard drug linagliptin. In addition, these compounds maintained a stable conformational interaction with the binding sites of TGFβR-1, as shown in the molecular dynamic simulation trajectories analysis. The binding affinity and conformational interaction pattern demonstrated that these flavonoids may compete with TGF-β for TGFβR-1, thereby limiting the hyperactivation of TGF-β signaling and subsequent protection against kidney fibrosis. However, in the future, for the development of protective agents against kidney fibrosis, several practical concerns need to be addressed, including drug–drug interactions, side effects, overlapping safety issues, dosage, as well as futility restrictions, which prevent them from being used in certain situations. Therefore, subject to the successful resolution of the experimental issues, our selected flavonoid-based agents are expected to overcome all these major challenges and act as a major therapeutic option for the treatment of kidney fibrosis.

## Figures and Tables

**Table 1:** List of compound codes, PubChem CID, chemical name, two-dimensional structure, and control compounds with the best binding affinity and MMGBSA score of the selected five phytochemicals.

Code	PubChem CID	Chemical Name	Chemical Structure	Docking Scores (kcal/mol)	MMGBSA Scores (kcal/mol)
C-06	72,276	(-)-Epicatechin	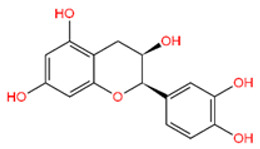	−13.585	−48.19
C-18	5,281,614	Fisetin	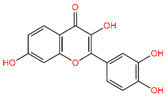	−13.177	−51.84
C-29	5,280,445	Luteolin	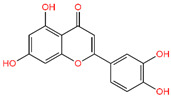	−10.506	−44.80
C-09	445,154	Resveratrol	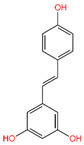	−8.552	−43.34
C-01	136,417	5-Hydroxy-3,6,7,8,3′,4′-hexamethoxyflavone	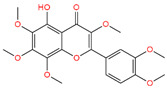	−7.808	−47.97
C-32	10,096,344	Linagliptin (control)	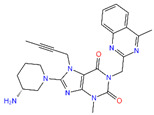	−9.074	−41.57

**Table 2:** Bonding interactions between the selected phytochemicals with TGFβR-1.

PubChem ID	Residues	Distance(Å)	Bond Category	Bond Type
CID-72276	Asp351	4.15	Hydrogen	Conventional Hydrogen
	Tyr249	5.64	Hydrogen	Conventional Hydrogen
	Glu245	4.82	Hydrogen	Conventional Hydrogen
	His283	4.08	Hydrogen	Conventional Hydrogen
	Ala350	5.36	Hydrophobic	Pi–Alkyl
	Leu260	4.98	Hydrophobic	Pi–Alkyl
	Ile211	4.63	Hydrophobic	Pi–Alkyl
	Ala230	4.57	Hydrophobic	Pi–Alkyl
	Val219	5.08	Hydrophobic	Pi–Alkyl
	Leu340	4.58	Hydrophobic	Pi–Alkyl
	Lys232	4.17	Hydrophobic	Pi–Cation
CID-5281614	Glu245	2.7	Hydrogen	Conventional Hydrogen
	Ala350	2.81	Hydrogen	Carbon–Hydrogen
	Ala230	4.13	Hydrophobic	Pi–Alkyl
	Leu340	4.71	Hydrophobic	Pi–Alkyl
	Leu260	4.92	Hydrophobic	Pi–Alkyl
	Ile211	2.76	Hydrophobic	Pi–Sigma
	Val219	2.68	Hydrophobic	Pi–Sigma
	Lys232	3.85	Hydrophobic	Pi–Cation
CID-5280445	Gly286	3.61	Hydrogen	Carbon–Hydrogen
	Val219	4.34	Hydrophobic	Pi–Alkyl
	Ala350	4.74	Hydrophobic	Pi–Alkyl
	Ala230	5.24	Hydrophobic	Pi–Alkyl
	Lys232	4.41	Hydrophobic	Pi–Cation
CID-445154	Ile211	4.78	Hydrophobic	Pi–Alkyl
CID-136417	Lys232	2.37	Hydrogen	Conventional Hydrogen
	Ser280	2.37	Hydrogen	Conventional Hydrogen
	Ala350	2.95	Hydrogen	Carbon–Hydrogen
	Ala230	4.57	Hydrophobic	Pi–Alkyl

## Data Availability

Not applicable.

## Supplementary Materials

The following supporting information can be downloaded at: https://www.mdpi.com/article/10.3390/life12111764/s1, Table S1: Compound’s screening and ADMET prediction; Table S2: Final 30 screened compounds; Table S3: The Docking Score of 6 control drugs; Table S4: Part-1 Prime_mmgbsa and Part-2 Final selected Docking Score Data.
