# Supplementary material for: An In-Silico Identification of Potential Flavonoids against Kidney Fibrosis Targeting TGFβR-1
Source: Life (Basel). 2022 Nov 2;12(11):1764. doi: 10.3390/life12111764 (PMC9694304; doi:10.3390/life12111764)
Supplement: Supplementary file 1 [file life-12-01764-s001.zip › Table S3. The Docking Score of 6 control drugs.pdf]

The Docking Score Data of the Targeting 6 control drug after ADMET screening.

S.N.	Pubchem CID	Compound Name	Docking Score
01	10096344	Linagliptin	-9.074
02	11450633	Alogliptin	-8.716
03	3961	Losartan	-6.971
04	6918537	Vildagliptin	-6.963
05	4369359	Sitagliptin	-6.667
06	11243969	Saxagliptin	-5.327
