# Supplementary material for: An In-Silico Identification of Potential Flavonoids against Kidney Fibrosis Targeting TGFβR-1
Source: Life (Basel). 2022 Nov 2;12(11):1764. doi: 10.3390/life12111764 (PMC9694304; doi:10.3390/life12111764)
Supplement: Supplementary file 1 [file life-12-01764-s001.zip › Table S4. Part-2 Final selected Docking Score Data.pdf]

The Docking Score Data of the Selected Compounds after ADMET and Chemical based Screening:

S.N.	Pubchem CID	Compound Name	Docking Score
01	72276	(-)-Epicatechin	-13.585
02	439533	Taxifolin	-13.509
03	5281614	Fisetin	-13.177
04	9064	Cianidanol	-12.994
05	5281672	Myricetin	-12.532
06	440735	Eriodictyol	-11.951
07	5281807	Puerarin	-11.884
08	6B8Y - crystalized_ligand		-11.880
09	5281612	Diosmetin	-11.837
10	5281670	Morin	-10.925
11	5281803	Pratensein	-10.616
12	5280445	Luteolin	-10.506
13	5281654	Isorhamnetin	-10.441
14	5280343	Quercetin	-10.330
15	5281616	Galangin	-10.321
16	31553	Silibinin	-10.311
17	5280863	Kaempferol	-10.035
18	5281702	Tricin	-9.955
19	5280443	Apigenin	-9.703
20	932	5,7-Dihydroxy-2-(4-hydroxyphenyl)chroman-4-one	-9.676
21	5281607	Chrysin	-9.660
22	5281708	Daidzein	-9.325

23	5281605	Baicalein	-9.258
24	445154	Resveratrol	-8.552
25	5317750	Glycitein	-8.368
26	5280961	Genistein	-7.992
27	136417	5-Hydroxy-3,6,7,8,3',4'-hexamethoxyflavone	-7.808
28	72344	Nobiletin	-7.355
29	5280373	Biochanin A	-7.302
30	5280460	Scopoletin	-6.940
31	68077	Tangeretin	-5.386
